# Anomalous Left Circumflex Artery Originating From Right Coronary Cusp as Culprit Vessel in ST-Elevation Myocardial Infarction (STEMI)

**DOI:** 10.7759/cureus.66230

**Published:** 2024-08-05

**Authors:** Abhishek Vadher, Nikhale Malik, Kurian Pannikottu, Ashok Kondur, Sujata Kambhatla

**Affiliations:** 1 Internal Medicine, Garden City Hospital, Garden City, USA; 2 Cardiology, Garden City Hospital, Garden City, USA

**Keywords:** inferior wall myocardial infarction, cardiac troponin, troponin, coronary vessel anomaly, st-elevation myocardial infarction (stemi)

## Abstract

Congenital coronary artery anomalies are rare. The most common anomalous variation is Anomalous Left Circumflex Artery (ALCx) which is a congenital anomaly. ALCx usually originates from the right sinus of Valsalva or as a proximal branching of the Right Coronary Artery (RCA). The clinical presentation has a spectrum which varies from asymptomatic presentation to angina or myocardial infarction with no atherosclerotic lesion due to kinking/compression of the vessel to ST-segment elevation myocardial infarction (STEMI) due to atherosclerotic occlusion.

A 45-year-old female with a past medical history of hypertension, hyperlipidemia, type 2 diabetes, tobacco abuse, and a history of ischemic stroke, presented to the hospital due to chest pain. Electrocardiogram revealed inferior ST-elevation myocardial infarction (STEMI) and the patient was taken to the catheterization lab. Angiography revealed 90% stenotic Left Circumflex Artery (LCx) which was anomalous, arising from the right coronary cusp, whereas other coronary arteries were diffusedly atherosclerotic. A drug-eluting stent was placed in the ALCx reducing the stenosis from 90% to 0% and the patient was discharged in a stable condition on dual antiplatelet therapy and statin with plans for possible coronary artery bypass graft due to multivessel disease (severe diffuse disease of LAD, 90% mid-RCA stenosis, 80% proximal RCA stenosis). The patient was eventually lost to follow-up.

Typically, anomalous LCx originating from RCA is benign, but there are many cases where there is myocardial infarction or sudden cardiac death due to acute angle take-off at the origin site. This anatomical variation is more important during cardiac surgeries because during valve replacement surgeries, there are cases of ALCx compression resulting in myocardial infarction. Our patient developed STEMI secondary to atherosclerotic stenosis in anomalous LCx. Based on her diffuse atherosclerotic disease and risk factors, it is likely that her anomalous anatomy did not cause her atherosclerotic disease. Overall, clinicians should remain vigilant for these anatomic abnormalities in their practice.

## Introduction

Congenital coronary artery anomalies are rare, and the overall incidence of all anomalies is about 0.2-1.3% [1]. The most common variation is Anomalous Left Circumflex Artery (ALCx) [2,3]. Generally, an ALCx originates from the right sinus of Valsalva or as a proximal branching of the Right Coronary Artery (RCA) [4]. The ALCx has three subtypes: Type I, which has a separate ostium for RCA and Left Circumflex Artery (LCx), Type II, which has a common ostium in the right sinus for the RCA and LCx, and Type III, in which the LCx arises as a branch of the proximal RCA [5]. The clinical presentation may vary from asymptomatic, to angina or myocardial infarction with no atherosclerotic lesion, to ST-segment elevation myocardial infarction (STEMI) due to atherosclerotic occlusion. Cardiac symptoms may develop due to the kinking/compression in the retroaortic course (malignant course) without any atherosclerosis in coronary vessels. In this case report, we present a case of ALCx which originated from the right coronary cusp which was the culprit artery in STEMI (type II).

## Case presentation

**Figure 1:**
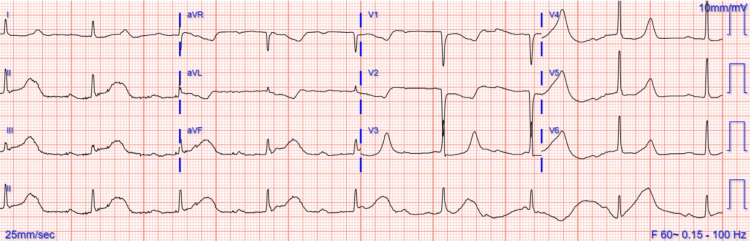
Electrocardiogram of our patient demonstrating ST-segment elevations in leads II, III, aVF, indicative of an inferior myocardial infarction.

A 45-year-old female with a past medical history of hypertension, hyperlipidemia, type 2 diabetes, tobacco abuse, and a history of ischemic cerebellar stroke, presented to the hospital due to chest pain for about 3 hours. She woke up in the morning, with intermittent, achy, substernal chest pain radiating to the left arm and rated as 10/10 in intensity. She also complained of diaphoresis and nausea, she denied palpitations, shortness of breath, and vomiting. Electrocardiogram (EKG) upon arrival to Emergency showed ST-elevations in leads II, III, and aVF suggesting inferior wall ischemia (Figure 1). Initial troponins were 26 ng/L. ST-segment elevation in voltages was more in lead II as compared to lead III which suggested that the inferior wall was supplied by LCx and LCx was the culprit artery. The patient was taken to the cath lab for emergent Left Heart Catheterization (LHC). The comprehensive metabolic panel was within normal limits. The lipid panel revealed cholesterol of 271 mg/dL, high-density lipoprotein (HDL) 34.6 mg/dL, low-density lipoprotein (LDL) 165 mg/dL, and triglycerides 507 mg/dL.

**Figure 2:**
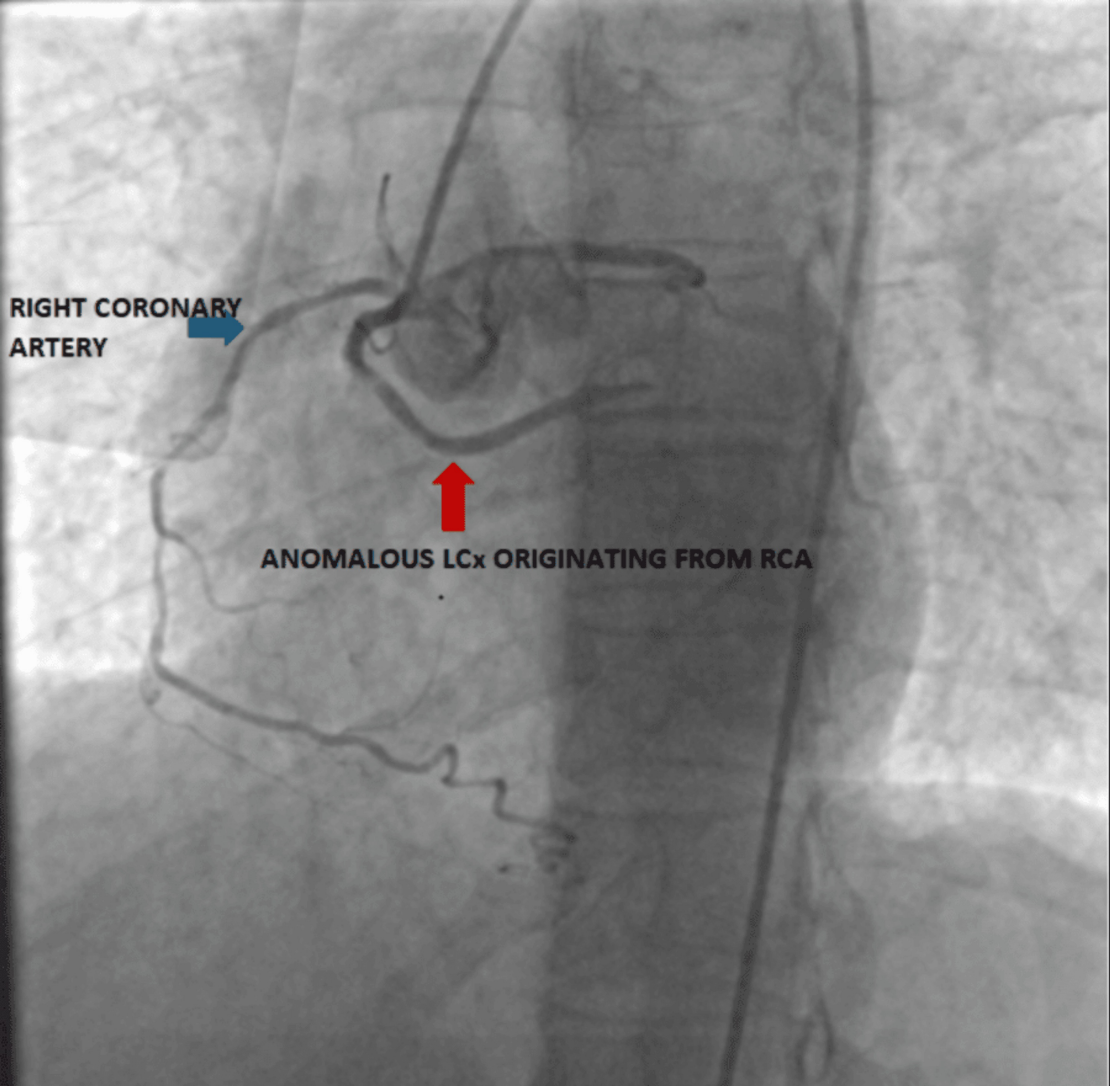
Red arrow shows the anomalous LCx originating from the right coronary cusp

Access was obtained via the right femoral artery. LHC demonstrated 90% stenosis of the Left Circumflex Artery (LCX) with Thrombolysis in Myocardial Infarction (TIMI) III flow, 90% mid-RCA stenosis with TIMI III flow, and 80% proximal RCA stenosis with TIMI III flow. LCX was noted to be arising from the right coronary cusp (Figure 2) (Video 1).

**Video 1:** Left heart cath showing anomalous LCx from the right coronary cusp

A drug-eluting stent (Onyx 3mm x 22mm) was placed to proximal mid-LCX improving occlusion from 90% to 0%. The patient was subsequently placed on dual anti-platelet therapy with Aspirin 81mg daily and Ticagrelor 90mg twice daily, as well as Atorvastatin 40mg daily.

Following the procedure, the patient became hemodynamically unstable with a 2:1 AV block requiring transfer to the ICU and she was placed on a Dopamine drip in addition to post-STEMI protocol medications. The post-procedural 2:1 AV block was transient and quickly resolved. Following a short stay in the ICU, the patient was discharged in a stable condition with outpatient cardiology follow-up. The patient is currently scheduled for a coronary artery bypass graft to be performed at a later date due to multivessel disease (severe diffuse disease of LAD, 90% mid-RCA stenosis, 80% proximal RCA stenosis).

## Discussion

One of the most common anomalies of the coronary arteries is a variant LCx originating from the sinus of RCA [6]. The prevalence of this anomaly was 0.37% in a study by Cleveland Clinic which consisted of 126,595 US patients’ arteriographies [6]. The prevalence is 0.67% in other US studies consisting of 2,996 patients and 0.17% in 16,573 patients from Turkey [3]. There are many other coronary artery anomalies and many of these are associated with sudden cardiac death. Other coronary artery anomalies that have been studied in detail are the high origin of coronary artery where the artery originates above the sinotubular junction on the aorta, left main coronary artery branching from RCA, or the RCA branching from the proximal left LCA [7]. Left coronary artery (LCA) branching from the proximal region of RCA shows the greatest incidence of sudden cardiac death [8-10]. It is suspected that myocardial ischemia leading to sudden cardiac death is related to arterial compression or structural variations that limit blood supply to the heart [11]. Occasionally, the anomalous arteries move between the pulmonary artery and aorta intraarterially and rarely arise intramurally within the aortic wall [7,12]. If an anomalous coronary artery causes a reduction in myocardial perfusion, the patient is at a very high risk of sudden cardiac death.

If LCx arises from RCA, typically it will move in a retro aortic manner, looping around the aorta posteriorly towards the left lateral wall of the heart to supply the myocardium [7]. In this anomaly, there is no intra-arterial course and is typically a benign variant [12]. There are few case reports of myocardial infarction in this anomaly, which is likely related to acute angle take-off or atypical chest pain luminal narrowing, or significant compression. There are case reports where there is a sudden cardiac arrest in which there was no evidence of obstructive atherosclerosis, but patients had anomalous LCx artery arising from the right sinus of Valsalva [13], nonetheless in most cases it is described as an incidental finding after Left Heart Cardiography done for another indication [14-15]. Large studies about this variation did not report an increase in the incidence of angina, myocardial infarction, and sudden cardiac death, and is described as a benign anomaly [6,8,16]. Clinically, this variation is important during cardiac surgery, because during valve replacement surgery, there are cases of ALCx compression resulting in infarction [17-18].

With regards to our patient, she developed STEMI secondary to ischemia from the ALCx. It has been postulated as to whether an ALCx may increase a patient’s atherosclerotic cardiovascular disease (ASCVD) risk due to the retroaortic flow and high angulation leading to increased stress on the anomalous artery. A retrospective cohort study done by Mohsen et al. found that the presence of an ALCx arising from the right coronary cusp did not lead to an increase in Major Adverse Cardiovascular Events (MACEs) with no increase in risk for atherosclerotic disease [16]. This is consistent with our patient, as her concomitant coronary artery disease in multiple other vessels demonstrates that her atherosclerotic disease was not specific to her ALCx. This is further supported by the number of risk factors our patient had including hypertension, dyslipidemia, type 2 diabetes, and a history of ischemic stroke.

Though the presence of an ALCx may not be indicative of increased ASCVD risk, a lack of awareness of these anomalies may greatly impact patient outcomes for patients requiring cardiac surgery. For instance, patients getting aortic valve replacements while having an unknown ALCx may be at risk for suture ligation of the vessel or compression by a prosthetic valve [19].

## Conclusions

With this case report we highlight a rare case of ALCx arising from the right coronary sinus as the culprit vessel for STEMI. We aim to increase awareness of these anomalies so that clinicians can make appropriate diagnoses and avoid potential life-threatening complications during future interventions. We suggest that there is a strong need for enhancing the education and training of cardiologists and cardiac surgeons in recognizing and managing coronary artery anomalies. Areas of future research include more comprehensive screening of coronary arteries for anomalies in patients with unexplained cardiac symptoms.
